# Two isoforms of the RAC-specific guanine nucleotide exchange factor TIAM2 act oppositely on transmission ratio distortion by the mouse *t*-haplotype

**DOI:** 10.1371/journal.pgen.1007964

**Published:** 2019-02-28

**Authors:** Yves Charron, Jürgen Willert, Bettina Lipkowitz, Barica Kusecek, Bernhard G. Herrmann, Hermann Bauer

**Affiliations:** 1 Department of Developmental Genetics, Max Planck Institute for Molecular Genetics, Berlin, Germany; 2 Institute for medical Genetics, Campus Benjamin-Franklin, Charité –University Medicine Berlin, Berlin, Germany; University of Illinois, UNITED STATES

## Abstract

Transmission ratio distortion (TRD) by the mouse *t*-haplotype, a variant region on chromosome 17, is a well-studied model of non-Mendelian inheritance. It is characterized by the high transmission ratio (up to 99%) of the *t*-haplotype from *t*/+ males to their offspring. TRD is achieved by the exquisite ability of the responder (*Tcr*) to trigger non-Mendelian inheritance of homologous chromosomes. Several distorters (*Tcd1-Tcd4*), which act cumulatively, together promote the high transmission ratio of *Tcr* and the *t*-haplotype. Molecularly, TRD is brought about by deregulation of Rho signaling pathways via the distorter products, which impair sperm motility, and the *t*-sperm specific rescue of sperm motility by the responder. The *t*-sperm thus can reach the egg cells faster than +-sperm and fertilize them. Previously we have shown that the responder function is accomplished by a dominant negative form of sperm motility kinase (SMOK^TCR^), while the distorter functions are accomplished by the Rho G protein regulators TAGAP, FGD2 and NME3 proposed to function in two oppositely acting pathways. Here we identify the RAC1-specific guanine nucleotide exchange factor TIAM2 as modifier of *t*-haplotype TRD. *Tiam2* is expressed in two isoforms, the full-length (*Tiam2l*) and a short transcript (*Tiam2s*). *Tiam2s* expression from the *t*-allele is strongly increased compared to the wild-type allele. By transgenic approaches we show that *Tiam2s* enhances *t*-haplotype transmission, while *Tiam2l* has the opposite effect. Our data show that a single modifier locus can encode different gene products exerting opposite effects on a trait. They also suggest that the expression ratio of the isoforms determines if the outcome is an enhancing or a suppressive effect on the trait.

## Author summary

Exceptions to Gregor Mendel’s rules of inheritance have attracted geneticists over many decades. One of the best-studied cases in mammals is the extremely high transmission rate (up to 99%) of a chromosomal variant in mouse, called the *t*-haplotype, from *t*/+ heterozygous males to their offspring. Previous studies have revealed that non-equal inheritance of the homologous chromosomes is due to motility differences between sperm containing the *t*-haplotype or the +-chromosome. Several regulators of Rho small G protein signaling additively contribute to the motility defects, while a mutant protein kinase is able to rescue sperm motility. However, it does so only in sperm containing the *t*-haplotype enabling them to reach the egg cells faster than the competing +-sperm. In this work we show that *Tiam2*, a gene known to be involved in cell motility control and tumor metastasis, is expressed in two isoforms, which exert opposite effects on *t*-haplotype transmission. The relative amounts of the two transcripts can either promote *t*-haplotype transmission or act oppositely. Our study reveals a novel type of quantitative trait locus, which can switch between a promoting and an inhibiting state.

## Introduction

According to Mendel’s rules, diploid organisms transmit the two alleles of a gene located on the homologous chromosomes at an equal ratio to their offspring. However, exceptions to “fair” transmission of genetic material to the next generation have been observed in most domains of life, and one of the most illustrative examples in mammals is Transmission Ratio Distortion (TRD) caused by the *t*-haplotype of the mouse (the term TRD is also used for other types of non-Mendelian inheritance) [1–3]. The *t*-haplotype is a variant form of the proximal half of chromosome 17, found in ~10% of wild mice of the *Mus musculus* species. Males heterozygous for a complete *t*-haplotype (*t/+*), on a permissive wild-type background, transmit the *t*-chromosome to an abnormally high proportion (up to 99%) of their offspring, on the expense of the wild-type chromosome [4]. The *t*-haplotype differs from the wild-type by the presence of four large non-overlapping inversions, which strongly suppress meiotic recombination between wild-type and *t*-haplotype DNA ensuring co-transmission of all factors involved in TRD [5–8]. However, due to rare recombination events that gave rise to partial *t*-haplotypes, several factors involved in TRD have been genetically separated and could thus be distinguished [9, 10]. The TRD factors are all located in the *t*-haplotype region of chromosome 17, are expressed during spermatogenesis and affect sperm motility. They consist of several *t*-*complex-distorters* (*Tcd1-4*) acting in *trans* on the *t*-*complex-responder* (*Tcr*). Distorters are genetic variants with the ability to exert a quantitative effect on a genetic trait (quantitative trait loci); they additively promote the transmission of the *t*-haplotype. Lyon proposed that the distorters have a hazardous effect on the wild-type form of the responder, while *Tcr* protects against this effect [9, 11]. However, this rescuing effect of *Tcr* is overcome by excessive distorter activity resulting in male sterility of homozygous *t/t* mice.

Insight into the molecular mechanism causing TRD has been gained from the cloning of *Tcr* [12]. It encodes a mutant, dominant negative form of sperm motility kinase (SMOK), termed SMOK^Tcr^. This finding suggested that the distorter genes encode members of a signaling pathway controlling SMOK activity [1]. SMOK is deregulated by the additive action of the distorters and hyperactivated resulting in impaired sperm motility. All sperm are affected, but *t*-sperm is rescued by the dominant negative kinase SMOK^TCR^ resulting in TRD in favour of the *t*-haplotype [1]. Indeed, we have been able to isolate three genes with distorter activity, and have shown that each of them promotes *t*-haplotype transmission. They all act as regulators of Rho small G proteins and thus are components of signaling pathways. These are TAGAP, a GTPase activating protein [13], FGD2 a GDP/GTP exchange factor (GEF) [14], and the nucleoside diphosphate kinase NME3 [15]. Based on the action of each of these factors we proposed that they are involved in controlling two pathways, one activating and the other inhibiting SMOK [14]. The *t*-alleles encoding variants of these factors are altered in a way that the activating pathway is upregulated and the inhibitory pathway is downregulated. The combinatorial action of both pathways thus results in hyperactivation of SMOK.

Rho small G proteins are known to regulate a large variety of cellular processes, many associated with dynamic cytoskeleton reorganization, most prominent among them the directional cell movement via chemotaxis. Many regulators of Rho G proteins have been associated with tumor cell migration and metastasis [16].

Here we identify the guanine nucleotide exchange factor TIAM2 (T-cell lymphoma invasion and metastasis 2) as distorter. *Tiam2* encodes two transcript (*Tiam2l* and *Tiam2s*) and protein isoforms expressed in developing sperm. We show that the two isoforms have opposite effects on *t*-haplotype transmission. The short isoform *Tiam2s* is expressed at an enhanced level from the *t*-allele as compared to wild-type, and thus adjusted for promoting *t*-haplotype transmission.

## Results

### *Tiam2* is expressed in two isoforms showing differential expression patterns between the wild-type and the *t*-haplotype allele

**Fig 1:**
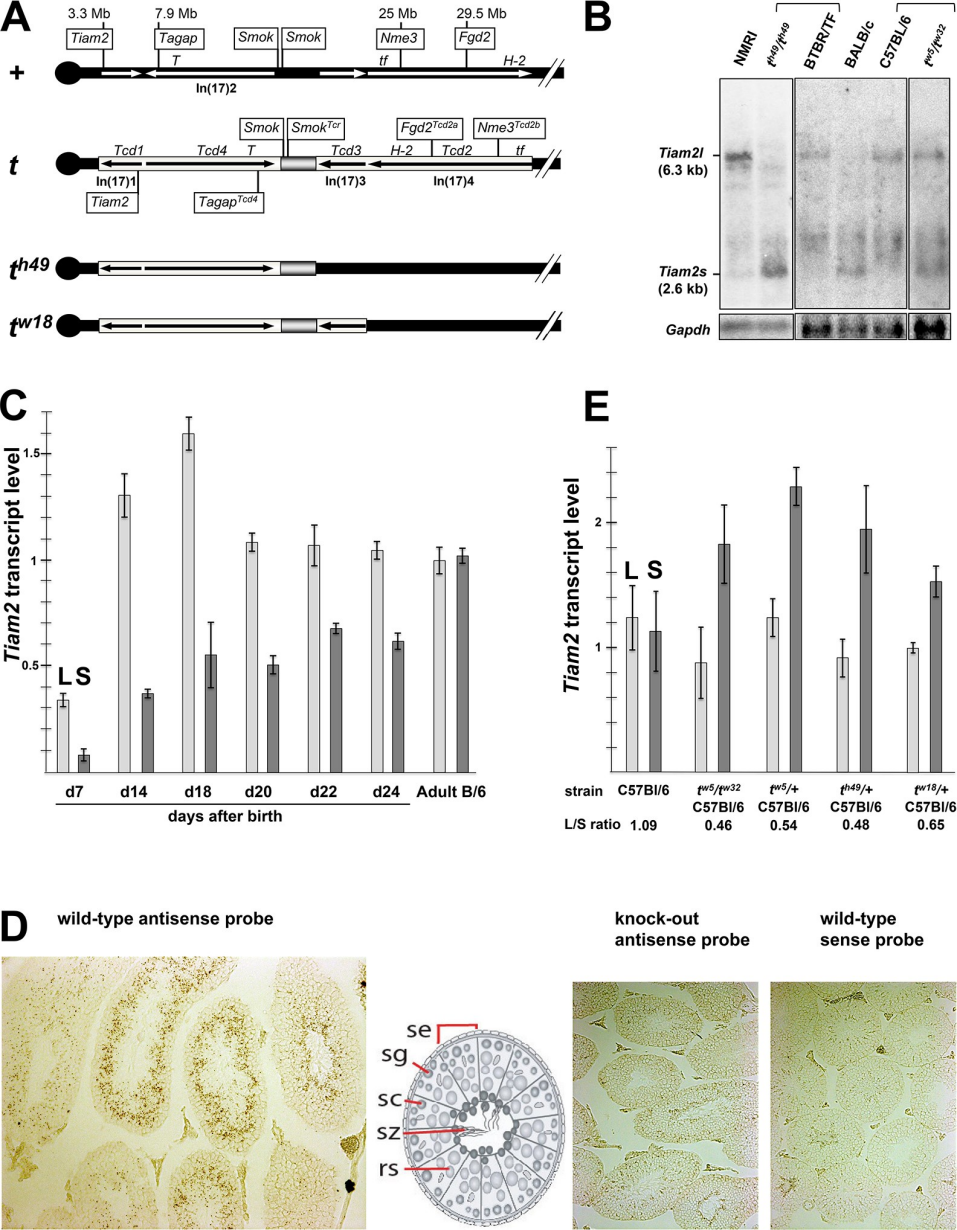
Two transcript isoforms of *Tiam2* are expressed in spermatogenic cells. (A) Schematic drawing of the wild type *t*-complex (+) and the *t*-haplotype (*t*). Genes with proven function in TRD are boxed; inversions are indicated by arrows. (B) Northern blot analysis of *Tiam2* expression in testes of wild-type and *t*-haplotype mice. (C) RT-qPCR analysis of *Tiam2* transcripts (L is *Tiam2l* and S is *Tiam2s*) in testes of C57BL/6 wild-type mice at day 7 to day 24 after birth reflecting the first round of spermatogenesis, and in adult mouse testis. (D) *in situ* hybridization with a *Tiam2* probe. (E) RT-qPCR analysis of *Tiam2* transcripts in testis RNA derived from C57BL/6 or from mice carrying *t* haplotypes on the C57BL/6 background; all values were calculated relative to *Tiam2l* expression in one C57BL/6 sample set to 1. Abbr.: Mb, Megabases; *T*, Brachyury; *tf*, tufted; *H-2*, mouse MHC complex; Inv(17)1 to Inv(17)4, Inversions 1 to 4 spanning the *t*-complex; se, Sertoli cell; sg, spermatogonium; sc, spermatocyte; sz, spermatozoa; rs, round spermatid.

The previous identification of three Rho small G protein regulators (*Tagap*, *Fgd2* and *Nme3*) as *t*-*complex-distorters* prompted us to search for new distorter candidate genes encoding Rho protein regulators in the *t*-haplotype region of chromosome 17. We identified the gene *Tiam2* (*T-cell lymphoma invasion and metastasis 2*) in the genetically defined *Tcd1* region close to *Tagap* as candidate (Fig 1A). *Tiam2* encodes a RAC1-specific guanine nucleotide exchange factor (GEF) [17, 18] and is a mammalian homologue of Drosophila SIF (*still life*) involved in synaptic growth [19]. Expression in testis and genetic variability between *t*- and wild-type alleles are critical criteria for a distorter candidate. Human and mouse *Tiam2* have previously been reported to be expressed in the testis, among other organs [17, 18]. We detected the long 6.3 kb *Tiam2* transcript (*Tiam2l*), described in mouse previously, by Northern analysis as a low level transcript in testicular RNA (Fig 1B). In addition, we identified a short transcript (*Tiam2s*) of 2.6 kb, which has not been described in mouse yet.

We confirmed *Tiam2s* by RT-PCR. We identified a 5’-region of *Tiam2s* mRNA, which is transcribed from intron 15 adjacent to and contiguous with exon 16, and therefore is not present in the mature *Tiam2l* transcript (S1 Fig); (for details see Methods). This data suggested that *Tiam2s* has its own transcriptional start site and therefore is controlled by a distinct promoter. We isolated a cDNA of *Tiam2s* comprising the 5’-untranslated region, starting in intron 15, exon 16 to 27 and an open reading frame of 579 amino acid residues. A short *TIAM2* transcript of 3.3 kb, termed *TIAM2S*, encoding the same part of the *Tiam2* open reading frame as mouse *Tiam2s*, has also been identified in human [18]. The Northern blot also reveals additional bands, which might represent alternative *Tiam2* transcripts that have been annotated in mouse genome databases. However, we have not been able to confirm the identity of these transcripts in testis.

We applied RT-qPCR to analyze the onset and course of expression of *Tiam2l* and *Tiam2s* in the testis of prepubertal C57BL/6 males during the first cycle of spermatogenesis (Fig 1C). Expression of both transcripts is detected already at the earliest time point examined, at day 7 after birth representing the pre-meiotic stages of spermatogenesis. Both *Tiam2l* and *Tiam2s* show an increase of expression from day 7 to day 18, the latter representing the postmeiotic stage of spermatogenesis. While *Tiam2l* expression remains at the same level at later stages, the expression of *Tiam2s* increases during late spermiogenesis and finally reaches the level of *Tiam2l* in the C57BL/6 adult gonad. The significance of these expression patterns is unclear.

The analysis of *Tiam2* expression by *in situ* hybridization of testis sections conforms with the qPCR results. The majority of transcripts were detected in the region of the seminiferous tubule, which harbours spermatocytes and spermatids (Fig 1D).

We investigated if the protein coding sequence of *Tiam2* expressed from the *t*-haplotype allele reveals sequence variations with respect to the wild-type gene, which might change the function of TIAM2. We derived cDNA fragments from testicular RNA of *t*^*h49*^/*t*^*h49*^ animals by RT-PCR and identified several single nucleotide polymorphisms (SNPs) listed in dbSNP data, and one *t*-specific SNP, which does not lead to an amino acid change (S1 Table). In conclusion, we did not detect any new mutations, which might alter the function of the TIAM2 proteins expressed by the *t*-allele.

Therefore we had a closer look at the abundance of the two transcript variants in *t* versus wild type (Fig 1B and 1E). We found that the relative levels of *Tiam2l* and *Tiam2s* transcripts identified by Northern blot analysis is reversed in *t*^*h49*^/*t*^*h49*^ as compared to the wild type genetic background on which this strain was maintained (BTBR/TF). Mice of the genotype *t*^*w5*^/*t*^*w32*^ also showed a higher transcript level of *Tiam2s* when compared to their wild type genetic background (C57BL/6). Thus the data suggest a strong cis-effect in setting the *Tiam2s* transcript level expressed from the *t* allele. Three out of four wild type strains analyzed showed a higher *Tiam2l* transcript level as compared to *Tiam2s*. However, we also found one strain, BALB/c, which shows stronger *Tiam2s* expression (Fig 1B).

We quantified the *Tiam2l* and *Tiam2s* transcript levels derived from the *t*-haplotype maintained on the inbred strain C57BL/6 in comparison to wild type C57BL/6 by qPCR (Fig 1E). The data show a strong increase of *Tiam2s* expression in *t*^*w5*^*/t*^*w32*^ (C57BL/6) as compared to C57BL/6, amounting to a change from approximately a 1:1 ratio of *Tiam2s* to *Tiam2l* in C57BL/6 to approximately 2:1 in *t*^*w5*^*/t*^*w32*^ (C57BL/6). A similar excess of Tiam2s expression was observed in testis RNA derived from three *t*/+ heterozygotes maintained on C57BL/6. The data demonstrate the enhanced expression of the *Tiam2s* transcript from the *t*-allele of *Tiam2*.

In summary, *Tiam2* encodes two transcripts, *Tiam2l* and *Tiam2s*, which are both expressed in differentiating germ cells in testis. From the *t*-allele, *Tiam2s* is more strongly expressed than *Tiam2l*, while the wild-type allele commonly shows equal or stronger expression of *Tiam2l*.

### Overexpression of *Tiam2l* reduces the transmission rate of the *t*^*h49*^-haplotype

Previously we have identified distorter candidates as *t*-haplotype gene variants with altered gene dosage, expression level or protein function [13–15]. We showed that a change in the expression level of the wild-type allele of a distorter by transgene constructs or knockout produces a quantitative effect on the transmission rate of a partial *t*-haplotype comprising *Tcr*. Thus, a statistically significant change of the *t*-haplotype transmission ratio identifies a distorter.

**Fig 2:**
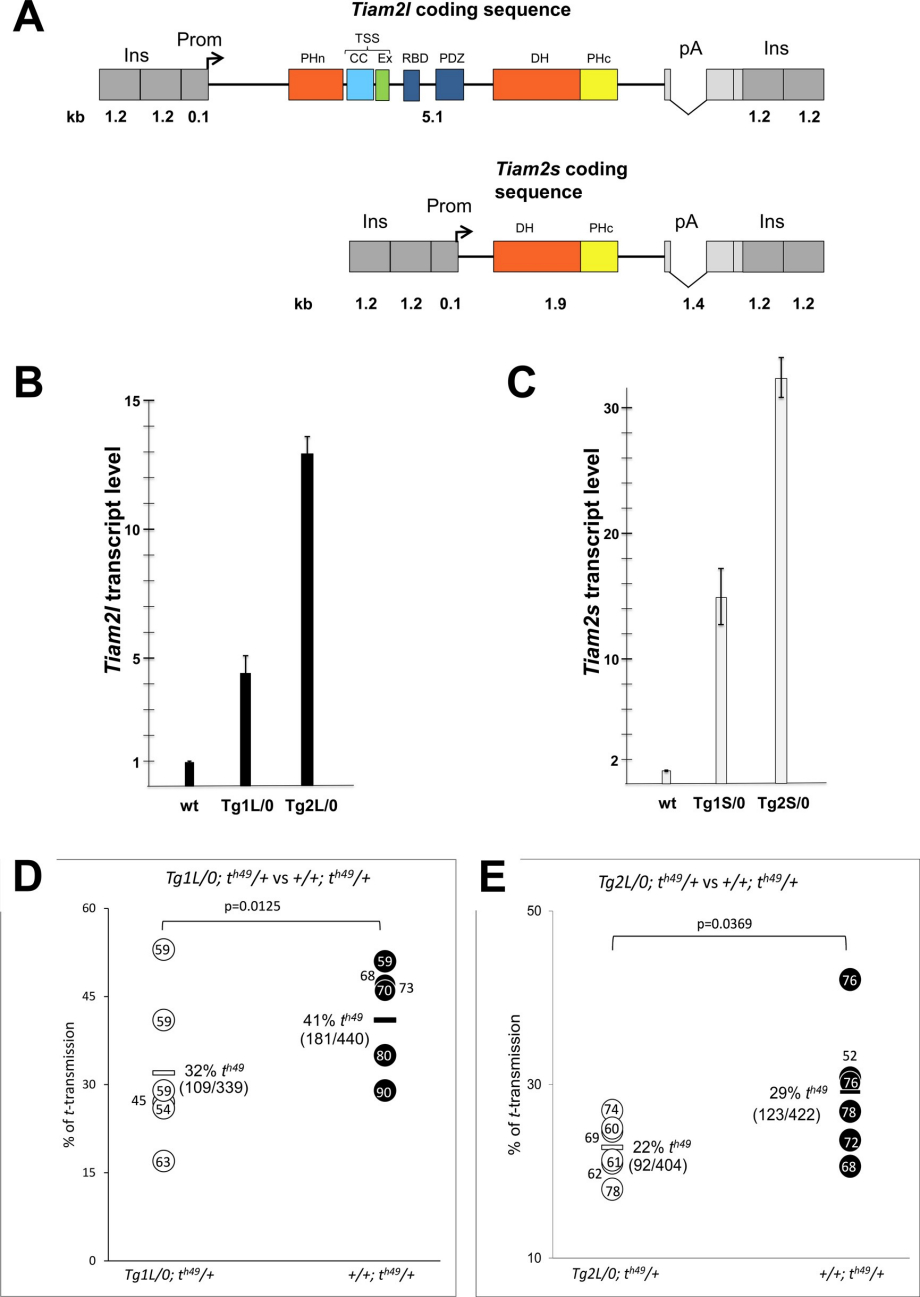
Transgenic overexpression of *Tiam2l* and *Tiam2s*. (A) Transgenic constructs for overexpression of *Tiam2l* and *Tiam2s* during spermatogenesis. The TIAM2 domain structure and abbreviations are according to [27] (B, C) RT-qPCR analysis verifying RNA expression of the transgenic constructs in the testis. (D, E) *t*-haplotype transmission ratio of TgL transgenic mice and non-transgenic control animals. Horizontal bars denote average *t*-transmission ratio (%); the number of *t* carrying out of the total offspring is given in brackets below. Dots indicate the number of offspring sired by individual males. See also S3 Table for total results. Abbr.:Ins, 2x chicken beta-globin insulator; kb, kilobases; pA, polyadenylation signals of rabbit beta-globin and SV40;Prom, promoter of the angiotensin converting enzyme.

In a first experiment, therefore we chose to increase the wild-type gene dosage of *Tiam2l* using a transgenic approach. We assessed if *in vivo* overexpression of *Tiam2l* in testis would alter the transmission ratio of the partial *t*-haplotype *t*^*h49*^. This *t*-haplotype contains the distorters *Tcd1*, *Tcd4* and *Tcr* (Fig 1A, and S2 Table). We generated a transgene construct expressing *Tiam2l* under control of the testis–specific *Ace* promoter in haploid sperm cells (Fig 2A) [20]. Several independent transgenic lines were generated. Quantitative PCR analysis of two lines Tg(Tiam2)C1L1Bgh and Tg(Tiam2)C1L2Bgh, abbreviated Tg1L and Tg2L respectively, indicated that the transgene construct was highly expressed in testis (Fig 2B). These two transgenic lines were crossed to animals carrying the *t*^*h49*^-haplotype. The transmission of the *t*^*h49*^ chromosome from transgenic (Tg/0; *t*^*h49*^/+) and non-transgenic (0/0; *t*^*h49*^/+) brothers to their offspring was analyzed (Fig 2D and 2E).

It is important to note that the transmission ratio of a particular *t*-haplotype strongly depends on the number of *t*-distorter loci encoded by the particular *t*-haplotype, and on the genetic background (S2 Table, [9, 21]). For instance, the partial *t*-haplotype *t*^*h49*^ containing *Tcd1* and *Tcd4*, but lacking *Tcd3* and *Tcd2*, has a lower transmission rate than *t*^*w18*^, which contains *Tcd1*, *Tcd4* and *Tcd3*, but not *Tcd2* [9]. However, this is only the case if both *t*-haplotypes are maintained on the same genetic background. The genetic background in fact has a tremendous impact on *t*-haplotype transmission [21, 22]. Since our *t*-haplotype strains and our transgenic lines are established and maintained on different genetic backgrounds, breeding them together always leads to mixed genetic backgrounds of the test animals. To make up for this genetic variation of the test males it is essential to compare the averaged transmission rate of several transgenic males with that of non-transgenic brothers. It also means that transmission data of a particular *t*-haplotype cannot be compared between different experiments, since the genetic background always varies.

Transgenic males of both lines transmitted *t*^*h49*^ at significantly lower rates than non-transgenic brothers (Tg1L: 32% *t*^*h49*^ offspring from transgenic, 41% *t*^*h49*^ offspring from non-transgenic males; P = 0.0125; Tg2L: 22% *t*^*h49*^ offspring from transgenic, 29% *t*^*h49*^ offspring from non-transgenic males; P = 0.0369). This result demonstrated that *Tiam2l* reduces *t*-haplotype transmission and acts as distorter in TRD.

### The heterozygous knockout of both *Tiam2* isoforms reduces the *t*-haplotype transmission rate

**Fig 3:**
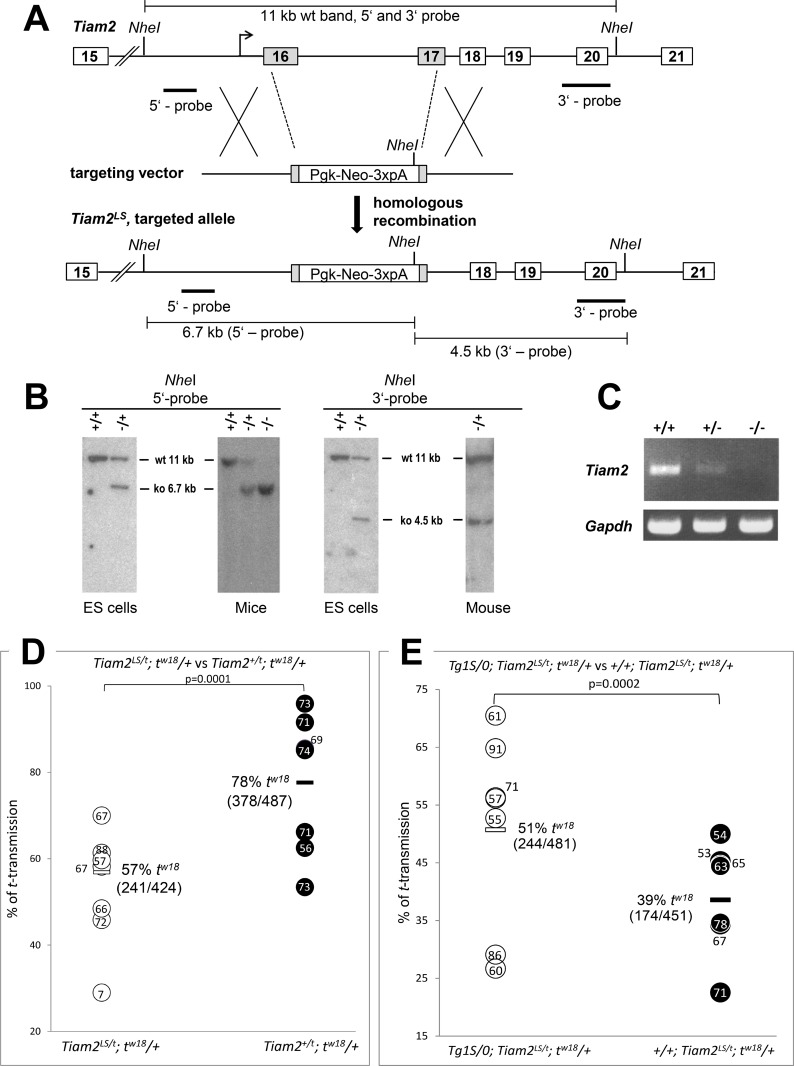
Targeted inactivation of the *Tiam2* gene. (A) Gene targeting strategy. *Tiam2* was inactivated by introducing a neomycin selection cassette deleting exons 16 and 17. Introns are depicted as line, exons as boxes and targeted exons as shaded boxes. (B) Confirmation of correct homologous recombination. Southern blot analysis of *Nhe*I digested genomic DNA of ES-cells and mice with the right (3’) and left (5’) external probes. (C) Loss of *Tiam2* transcription upon targeting of the *Tiam2* locus as determined by RT-PCR expression analysis of both *Tiam2l* and *Tiam2s* in wild-type (+/+), heterozygous (+/-) and homozygous (-/-) mutant testes using a primer pair detecting both transcripts (see S6 Table). (D, E) *t*-haplotype transmission ratio of *Tiam2*^*LS/t*^ males and *Tiam2*^*+/t*^ control animals, without (D) or carrying in addition the transgene construct Tg1S (E). Horizontal bars denote average *t*-transmission ratio (%); the number of *t* carrying out of the total offspring is given in brackets below. Dots indicate the number of offspring sired by individual males. See also S4 and S5 Tables for total results. Abbr.: wt, wild-type; ko, knockout; +, wild-type allele;—, knock-out allele; 3xpA, polyadenylation signal.

To confirm the function of *Tiam2* as distorter by a different approach, we generated a loss-of-function allele by targeting the wild-type allele of *Tiam2*. To completely abolish the expression of both the long and the short transcript isoforms, we replaced parts of exons 16 and 17 of the *Tiam2* gene by a Pgk::neo selection cassette (Fig 3A). This procedure should result in (i) premature termination of the long transcript upstream of the DH/PH catalytic domain, and (ii) complete lack of the *Tiam2s* transcript. The successful engineering of the mutant allele was checked by Southern blot analysis (Fig 3B). We introduced the targeted allele, *Tiam2*^*tm1Bgh*^, (called *Tiam2*^*LS*^ here) into the germ line and confirmed by RT-PCR that both *Tiam2* transcripts are lacking in the testis of males homozygous for the knockout allele (Fig 3C). To determine the effect of the mutant allele on TRD, mice heterozygous for *Tiam2*^*LS*^ were crossed to mice heterozygous for the partial *t*-haplotype *t*^*w18*^ carrying the *t*-allele of *Tiam2*. We tested transmission of the *t*^*w18*^ chromosome from brothers carrying either the *Tiam2* wild-type allele (*Tiam2*^*+/t*^*; t*^*w18*^*/+*) or *Tiam2*^*LS*^ (*Tiam2*^*LS/t*^*; t*^*w18*^*/+*) in *trans* to *t*^*w18*^, to their offspring (Fig 3D). While the control males (*Tiam2*^*+/t*^; *t*^*w18*^*/+*) transmitted *t*^*w18*^ to 78% of their offspring, the males lacking the *Tiam2* wild-type allele (*Tiam2*^*LS/t*^; *t*^*w18*^*/+*) transmitted the partial *t*-haplotype to just 57% of their offspring (P < 0.0001). This is the strongest change in the *t*-haplotype transmission ratio caused by a genetically manipulated allele we have observed so far, and strongly confirms that *Tiam2* acts as distorter.

### *Tiam2s* and *Tiam2l* have opposite effects on *t*-haplotype transmission

Though the knockout of both *Tiam2* isoforms demonstrated and confirmed the strong effect of *Tiam2* on TRD, the fact that the overexpression of *Tiam2l* and the knockout of *Tiam2* both reduced the *t*-haplotype transmission rate was puzzling. Since *Tiam2l* negatively affects *t*-haplotype transmission, the reduction of the *Tiam2l* gene dosage by the knockout should have increased the transmission rate of the *t*-haplotype. Since the opposite was the case, this suggested that the reduction of *Tiam2s* prevails against the reduction of *Tiam2l*. If that holds true, the overexpression of *Tiam2s* should enhance the *t*-haplotype transmission rate.

To test this assumption we generated transgenic lines expressing *Tiam2s* from a transgene construct under control of the testis-specific *Ace*-promoter (Fig 2A) [20]. We confirmed transgene expression in the testis by RT-qPCR (Fig 2C). We crossed two transgenic lines with mice carrying either the *t*^*h49*^-haplotype, showing a low transmission rate, or the *t*^*w18*^-haplotype showing high TRD in our stocks (S2 Table). The transgenic line Tg(Tiam2)C11S1Bgh (abbreviated Tg1S) showed a small increase of the *t*^*h49*^ transmission rate (3%, from 18% to 21%), as compared to non-transgenic brothers. However, this difference was statistically not significant. The transgenic line Tg(Tiam2)C11S2Bgh (abbreviated Tg2S) increased the high transmission rate of *t*^*w18*^ (82%) by only 1%, which is again statistically not significant (S3 Table).

We reasoned that the combined *Tiam2s* transcripts expressed from the *t*-haplotype and the wild-type allele together might render the sperm unresponsive to a further increase coming from the transgenic allele. Therefore we decided to sensitize the sperm by decreasing the *Tiam2* gene dosage via the *Tiam2*^*LS*^ knockout allele and test the effect of transgenic overexpression of the *Tiam2s* isoform on *t*-haplotype transmission in the context of *Tiam2* heterozygosity. We generated mice carrying the *Tiam2 t*-allele of the *t*^*w18*^-haplotype and the *Tiam2*^*LS*^ knockout allele on wild-type chromosome 17, and tested the transmission rate of *t*^*w18*^ from males carrying no transgenic construct or alternatively in addition Tg1S. While non-transgenic animals carrying *Tiam2*^*LS*^ (*+/+; Tiam2*^*LS/t*^*; t*^*w18*^*/+*) showed 39% transmission of the *t*^*w18*^-haplotype in this experiment, the presence of the *Tiam2s* expressing transgene (*Tg1S/0; Tiam2*^*LS/t*^*; t*^*w18*^*/+*) enhanced the *t*^*w18*^ transmission to 51% (Fig 3E). The difference is statistically highly significant. This data proves that *Tiam2s* indeed enhances *t*-haplotype transmission.

The combined data show that *Tiam2l* and *Tiam2s* exert opposite effects on *t*-haplotype transmission. *Tiam2l* reduces the transmission rate of a *t*-haplotype, while *Tiam2s* enhances it.

## Discussion

In this work we have shown that the *Tiam2* gene expresses two transcript isoforms in testis, *Tiam2l* and *Tiam2s*, which encode proteins that both exert quantitative but opposite effects on *t*-haplotype transmission. The two transcript isoforms not only show opposite effects, they also show opposite preferential expression patterns from the wild-type as compared to the *t*-haplotype allele. The expression of the short isoform was much stronger from the *t*-haplotype allele than from the wild type allele maintained on the same genetic background. The *Tiam2l/Tiam2s* transcript ratio in the inbred strain C57BL/6 in this study was measured as approximately 1.1, while in *t*^*w5*^*/t*^*w32*^ males maintained on the same C57BL/6 strain it was approximately 0.5. Several *t/+* heterozygotes maintained on the same C57BL/6 background also showed about twofold higher *Tiam2s* expression as compared to *Tiam2l*. These findings reveal a strong *cis* regulation effect on the *Tiam2s* promoter of the *t* allele. The wild-type strains examined in this report show a wide range of *Tiam2l/Tiam2s* transcript ratios estimated based on Northern signals suggesting strong differences in the control of wild-type *Tiam2* alleles. These observations are in good agreement with the reported variability of *t*-haplotype TRD on different genetic backgrounds [21, 22]. For instance, the transmission ratio of the *t*^*0*^ haplotype crossed to different strains was found to vary between 34% and 94% [22].

Together these observations provide direct insight into the genetic basis of competition between the *t*-haplotype and wild-type distorter alleles in promoting or counteracting *t*-haplotype transmission, respectively.

**Fig 4:**
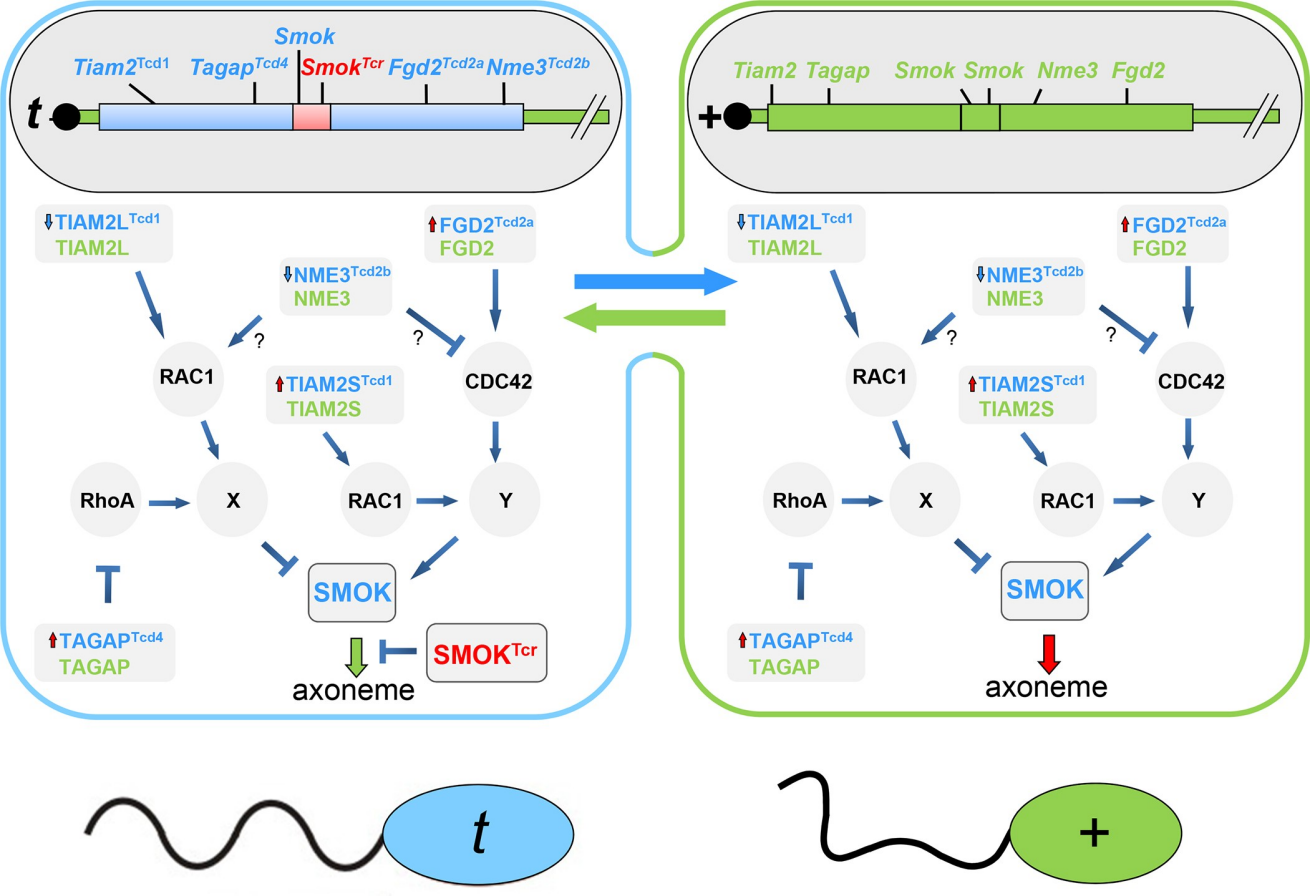
Molecular model of transmission ratio distortion comprising the opposing roles of TIAM2L and TIAM2S. Schematic drawing representing haploid sperm cells of a syncytium of a *t/+* heterozygous male undergoing spermatogenesis, the blue cell containing the *t*-haplotype expressing *Smok*, *Smok*^*Tcr*^ and the *t*-distorter alleles, the green cell carrying the wild-type *t*-complex expressing *Smok* and wild type distorter alleles. While the distorter products are exchanged between the cells (i.e. they act in *trans*), and together impair sperm motility, the SMOK proteins and SMOK^TCR^ are retained within the cell carrying the gene leading to a phenotypic difference of the spermatozoa, such that *t*-sperm (blue) are functionally rescued and show normal motility (indicated by a wavy tail) while +-sperm (green) remain impaired. Thus offspring of the *t/+* male preferentially inherit the *t*-haplotype. For details of the molecular model see the text. Red upward pointing arrows at distorter proteins indicate up-regulation, blue down-pointing arrows down-regulation; the green down-pointing arrow to the axoneme symbolizes rescued, the dark-red down-pointing arrow impaired flagellar motility.

Previously, we have identified three distorters: the Rho-GAP TAGAP, the Rho-GEF FGD2, and the nucleoside diphosphate kinase NME3. The former two are encoded by hypermorphic alleles in the *t*-haplotype, the latter by a hypomorphic allele. We have argued that TAGAP and FGD2 act on two different Rho signaling pathways, which exert opposite effects on SMOK, a kinase thought to control sperm motility downstream of the distorters [14]. According to our model TAGAP regulates an inhibitory and FGD2 an activating pathway controlling SMOK. The *t*-haplotype alleles enhance the Rho inhibitor TAGAP in the inhibitory pathway, and the Rho activator FGD2 in the activating pathway, resulting in parallel in reduced inhibition and enhanced activation of SMOK by the two pathways (Fig 4). Since a reduction of NME3 function promotes *t*-transmission, we have suggested that NME3 might either inhibit the activating pathway or activate the inhibitory pathway. The overall outcome is strong deregulation of SMOK causing impairment of sperm motility.

How does *Tiam2* fit into this model? The fact that *Tiam2l* reduces *t*-haplotype transmission, argues that *Tiam2l* is an activator of the inhibitory pathway, and therefore supposedly acts epistatically to *Tagap* (Fig 4). Since the expression of *Tiam2s* from the *t*-allele is enhanced and promotes *t*-transmission we argue that *Tiam2s* up-regulates SMOK, either through the same pathway as *Fgd2* or via a different, synergistic pathway. Thus *Tiam2* acts in parallel on both the activating and the inhibitory pathway.

In our previous TRD model we have been uncertain about the Rho G proteins controlled by TAGAP, FGD2 and NME3. The situation is more clear-cut with respect to TIAM2. TIAM2L has been shown to bind and regulate RAC1 [17], which puts the latter downstream of TIAM2L in the inhibitory pathway of our TRD model. FGD2 has been shown to bind and regulate CDC42 [23]. Therefore, we suggest that CDC42 might represent the Rho G protein of the activating pathway. We have shown that in vitro TAGAP is a GAP of RHOA [14]. Thus, provisionally we include RHOA in the inhibitory pathway. However, so far we have no additional evidence for an involvement of RHOA in TRD. GAPs show some promiscuity with respect to choice of Rho target proteins. Therefore it is not excluded that in sperm TAGAP might act through a different Rho GTPase.

Altogether, the reduced inhibition of the inhibitory pathway combined with the enhanced activation of the activating pathway(s) by the *t*-distorters causes hyperactivation of SMOK resulting in impaired motility of spermatozoa. The dominant negative kinase SMOK^TCR^, the protein product of *Tcr*, is able to counterbalance this hyperactivation and to rescue sperm motility. However, due to restriction of SMOK^TCR^ to the cells of origin (the ones carrying the *t*-haplotype) this happens exclusively in *t*-sperm [24]. The latter reach the egg cells faster and fertilize them, which results in preferential transmission of the *t*-haplotype.

How can TIAM2L and TIAM2S have opposite effects on TRD? Both contain the DH/PHc domain necessary and sufficient for RAC activation in vitro [17]. Additional domains and motifs are necessary for specific cellular localization and activities. It has been shown that the PHn and TSS domains present in TIAM2L but not in TIAM2S are necessary and sufficient for membrane localization [25]. In addition, recent crystal structure, binding and mutational analyses showed that the PHnCCEx (TSS) domain of TIAM2L mediates dual binding to membranes and signaling proteins [26, 27]. In sperm, TIAM2l might therefore activate RAC1 at the plasma membrane of spermatozoa, while TIAM2S might act in a different cellular compartment, for instance at the axoneme. In summary, it is quite likely that the two isoforms interact with different scaffold proteins and control signaling in different subcellular domains and compartments.

TIAM2 and its homologue TIAM1 act as RAC GTPases in cell motility, and are involved in cancer cell invasion and metastasis [28, 29]. Aberrant expression of TIAM2S in hepatocellular carcinoma cells (HCC) promoted an invasive phenotype in xenografted tumors. Thus TIAM1 and TIAM2S were suggested to interact in HCC to exert their oncogenic effects [30]. As an alternative explanation for the distinct function of TIAM2S it has been proposed that cytosolic TIAM2S may bind directly to a downstream effector and bypass the catalytic step of GDP/GTP exchange to trigger its tumorigenic effect in HCC [30]. The p21-activated kinase interacting GEF αPIX has been shown to stimulate PAK activity by a GEF-independent mechanism [31]. Thus it is conceivable that TIAM2S in sperm might also act on an effector via a GEF-independent mechanism, not via RAC1.

In summary, both human TIAM2L and TIAM2S are involved in the control of cell motility and may act by different mechanisms and in different cellular compartments.

Our finding that TIAM2L and TIAM2S are involved in TRD presented here further underpins the important role of Rho signaling in the control of sperm motility, and strengthens our proposal that spermatozoa use the same control mechanism for directional movement as slow moving cells [32].

Previously we have assigned *Tagap* to the *Tcd1* region and, with reference to Lyon’s suggestion that *Tcd1* may split into two loci, *Tcd1a* and *Tcd1b*, we designated the *t*-allele of *Tagap Tagap*^*Tcd1a*^ [33]. However, more recent genome assembly data have positioned *Tagap* just proximal of *Brachyury* (*T*), and thus within inversion In(17)2. Therefore *Tagap* in fact maps to the distorter region *Tcd4* and its *t*-allele henceforth is designated *Tagap*^*Tcd4*^ (Fig 1A). In accordance with this reassignment the *t*-allele of *Tiam2* is a candidate for *Tcd1* (Fig 1A).

## Methods

### Ethics statement

Animal experiments were approved by the ethics committee of the LAGeSo Berlin (registration numbers G 0368/08, G 0247/13, T0215/04, T0054/10, T0279/16).

### Transcript and genomic analysis

We isolated total RNA using Trizol (Invitrogen), removed genomic DNA contamination using DNA digestion with the DNA-free kit (Ambion) and analyzed RNA quality by gel electrophoresis. We performed reverse transcription using 1 μg of testis RNA with the SuperScript reverse transcription system (Invitrogen) or M-MLV Reverse Transcriptase (Promega). We designed transcript specific RT-qPCR assays using publicly available design tools (Primer3, NCBI) avoiding cross reaction and strain specific SNPs. We carried out gene expression analysis by quantitative PCR (RT-qPCR) using a StepOnePlus Real-Time PCR System (Life Technologies) and Power SYBR Green PCR Master Mix (Promega). Normalization was done with *Gapdh* as reference gene which has been validated for constant expression during testis development [34]. Quantification was performed using the ΔΔCt method after confirming similar efficiencies of RT-qPCR assays [35]. Error bars indicate the standard error from technical replicates (Fig 1C). We tested the *Tiam2l/Tiam2s* expression levels using 3 biological replicates of C57BL/6, *t*^*w5*^*/+*, *t*^*w18*^*/+*, *t*^*h49*^*/+* and *t*^*w5*^*/t*^*w32*^, all maintained on the C57BL/6 inbred background. Relative expression levels were calculated with respect to the *Tiam2l* expression level obtained in one of the three C57BL/6 samples set to 1 (for primer pairs and PCR conditions see S6 Table).

We isolated poly(A)+ RNA from mouse testes using the Micro Fast Track system (Invitrogen) after Trizol total RNA purification and performed Northern blot analysis using the NorthernMax-Gly Kit (Ambion) according to the manufacturer´s instructions. Northern hybridization was performed using ExpressHyb solution (Clontech) according to the manufacturer’s instruction.

We performed *in situ* hybridization using RNAscope (Advanced Cell Diagnostics) reagents and protocols according to the manufacturer’s information.

We identified the transcript start of *Tiam2s* by 5’-RACE and RT-PCR. 5’-RACE experiments revealed products starting at exon 16. RT-PCR with primers hb853-hb851 (see S6 Table) located further upstream identified a short 5’-region of *Tiam2s* mRNA transcribed from intron 15 adjacent to and contiguous with exon 16 (S1 Fig). This transcript is robustly expressed in testis as shown on Fig 1C and 1E. Using the same 5’ primer (hb853) and a reverse primer (Tiam2_3’-UTR-24) in exon 27 we isolated and sequence verified a fully spliced *Tiam2s* cDNA containing the whole ORF encoding the PHn, TSS, RBD and PDZ domains, by RT-PCR. We expressed this ORF from the transgene constructs Tg1S and Tg2S. The ORF corresponds to the ORF encoded by human *TIAM2S*.

We examined the testis expression of other transcripts annotated in public databases with a transcriptional start site and first exon located in intron 15. We found transcripts NM_001286757 (3.2 kb) and NM_001286758 (2.6 kb) robustly expressed in brain, but not expressed or barely detectable in testis. Putative first exons indicated by CAGE-Seq data of testis https://www.ncbi.nlm.nih.gov/biosample/SAMN01943339 did not reveal *Tiam2* RT-PCR products with exon-spanning PCR-primers. Therefore we concluded that the 2.6 kb *Tiam2s* transcript identified in this work corresponds to human *TIAM2S*. It is the only robustly expressed testis transcript starting from a TSS and first exon located in intron 15 showing differential expression between the wild-type and *t*-allele.

### Gene targeting and transgenic constructs

To generate a knock-out of *Tiam2*, we targeted the *Tiam2* locus in CJ7 ES-cells [36] by introducing a *Pgk1* promoter/neomycin resistance gene/ triple-polyA (Pgk1-neoflox3xpA)—cassette [14] deleting large part of exons 16 and 17 (chr. 17, bases 3,502,894 to 3,505,764, in GRCm38.p4) as depicted in Fig 3A. To construct the targeting vector, we isolated the left- and right homology arms (2,064 bp and 3,065 bp respectively) of the targeting construct by PCR and cloned both arms on either side of the selection cassette, introducing a *Cla*I site for linearization at the end of the left arm. We electroporated CJ7 ES-cells with the linearized targeting construct and selected, isolated and analyzed clones according to standard procedures [37]. We identified correctly targeted ES-clones by Southern hybridization of *Nhe*I digested genomic DNA with the left 5´-probe (LP) and right 3´-probe (RP) (primers 5´-LP and 3´-LP, 837 bp probe fragment; primers 5´-RP1 and 3´-RP1, 919 bp probe fragment). Both probes detect a 11.235 kb *Nhe*I fragment in wild type which, upon successful targeting, is altered to a 6.670 kb fragment for the 5´-probe and a 4.478 kb fragment for the 3´-probe. The targeted allele is named *Tiam2*^*tm1Bgh*^ (according to official nomenclature; abbreviated *Tiam2*^*LS*^ in the text). Chimeras were generated by blastocyst injection of a gene-targeted *Tiam2*^*LS*^ ES-cell clone according to standard procedures and the line was established on a 129/SvEv background.

The transgenic construct Tg(Tiam2)C1LBgh (abbreviated Tg1L in the text) consists of the spermiogenesis specific promoter of the angiotensin converting enzyme (*Ace*) including the transcriptional start site (pos. -91 to +17) [20] followed by the complete open reading frame of wild-type *Tiam2l*, the rabbit β-globin polyadenylation (pA) signal including an intron [38] and the SV40 polyadenylation signal. We flanked this expression cassette by 2 copies of the chicken beta-globin insulator on both sides [39]. Tg(Tiam2)C1SBgh (abbreviated Tg1S) contained the *Tiam2s* coding region in the same promoter-polyA-insulator backbone as Tg1L (Fig 2A). Tg2L and Tg2S were constructed accordingly. Transgenic constructs were isolated from the vector backbone and purified for pronuclear injection of fertilized C57Bl/6J eggs or ES-cell transfection using standard procedures. We identified transgenic ES cells and mice by PCR (for primer sequences see S6 Table).

### Mice and genetics

We used the following *t*-haplotypes for analyses: *t*^*w18*^*/+*, *t*^*h49*^*/+*, *t*^*h49*^*/t*^*h49*^, *t*^*w5*^*/t*^*w32*^. *t*^*w5*^ and *t*^*w32*^ are naturally occurring complete *t*-haplotypes, *t*^*w18*^ and *t*^*h49*^ are partial *t*-haplotypes derived from rare recombination events between *t*- and a wild-type chromosome. To analyze the effects of transgenic constructs and of the knock-out allele on TRD we mated males carrying a *t*-haplotype and the *Tiam2*^*LS*^ allele or transgenic construct with outbred females (NMRI) as described in the text. Since *t*-haplotypes and *Tiam2* alleles were kept and generated on diverse genetic backgrounds closely related non-transgenic males, usually brothers served as controls. We genotyped embryos by PCR using the primers vil2-L and vil2-R (MGI Accession ID 3033374) for assessing the inheritance of the *t*-haplotypes *t*^*w18*^ and *t*^*h49*^. Statistical analysis of results was done using Chi square test (χ^2^) with Yates correction calculating the chi-square and the two-tailed P-value.

## Supporting information

S1 Fig *Tiam2s* transcription starts in intron 15, the putative start of translation is in exon 16. The start codon is underlined, arrows indicate primers used for RT-qPCR (see S6 Table).
(TIF)

S1 Table Sequence analysis of *Tiam2* transcripts from wild type versus *t*-haplotype. (DOCX)

S2 Table *t*-haplotypes. Structure of different complete and partial *t*-haplotypes and their transmission ratio distortion rates depending on the genetic background.
(DOCX)

S3 Table Transgenic overexpression of *Tiam2l* decreases *t^h49^* transmission (Fig 2D and 2E). (DOCX)

S4 Table Heterozygous loss of *Tiam2* function strongly reduces the transmission rate of *t^w18^* (Fig 3D). (DOCX)

S5 Table Transgenic *Tiam2s* overexpression strongly increases the transmission rate of *t^w18^* upon loss of the wild-type *Tiam2* allele (Fig 3E). (DOCX)

S6 Table Oligonucleotides. Primer sequences, PCR conditions and results for transcript—and genomic analyses, generation of transgenics and gene targeting constructs.
(DOCX)
