# Supplementary figures and images for: Two isoforms of the RAC-specific guanine nucleotide exchange factor TIAM2 act oppositely on transmission ratio distortion by the mouse t-haplotype
Source: PLoS Genet. 2019 Feb 28;15(2):e1007964. doi: 10.1371/journal.pgen.1007964 (PMC6394906; doi:10.1371/journal.pgen.1007964)

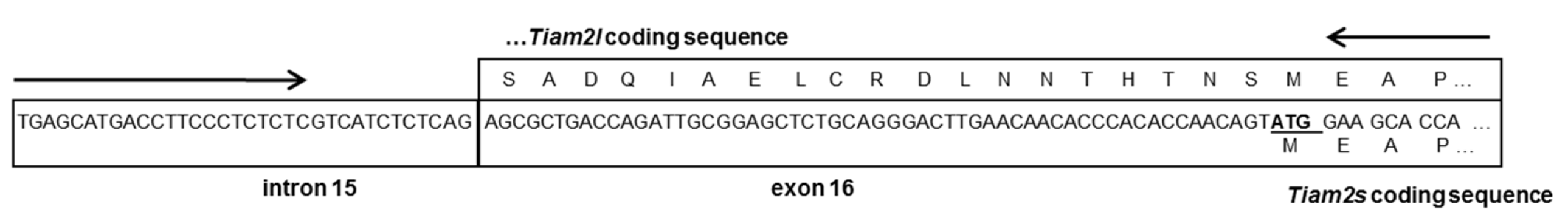

Supplement: S1 Fig — The start codon is underlined, arrows indicate primers used for RT-qPCR (see S6 Table). (TIF) [file pgen.1007964.s001.tif]
