# Supplementary material for: Two isoforms of the RAC-specific guanine nucleotide exchange factor TIAM2 act oppositely on transmission ratio distortion by the mouse t-haplotype
Source: PLoS Genet. 2019 Feb 28;15(2):e1007964. doi: 10.1371/journal.pgen.1007964 (PMC6394906; doi:10.1371/journal.pgen.1007964)
Supplement: S1 Table — (DOCX) [file pgen.1007964.s002.docx]

**Charron et al. Supplementary Table 1:** **cDNA sequence of *Tiam2l* (Transcript 201) from *t^h49^* in comparison with C57BL/6.**

| Exon | Sequence  C57Bl/6 - *t^h49^* | amino acid change | SNP |
| --- | --- | --- | --- |
| 1 | ATT - GTT | Silent (5’-utr ) | [rs33240796](http://www.ensembl.org/Mus_musculus/Variation/Explore?db=core;tl=WuT0GrYIcMGTG0hF-495910-71096584;v=rs33240796;vdb=variation;vf=6083659) |
| 8 | GGT - GGG | silent | [rs108882019](http://www.ensembl.org/Mus_musculus/Variation/Explore?db=core;tl=jYGqY7ingaNdslsb-495762-71083527;v=rs108882019;vdb=variation;vf=20198777) |
| 10 | GTG - GCG | V860A | [rs33661541](http://www.ensembl.org/Mus_musculus/Variation/Explore?db=core;tl=Rl2szG1OTs0RcYUO-495791-71084074;v=rs33661541;vdb=variation;vf=6482542) |
| 15 | CTG - CCG | L1077P | [rs33069377](http://www.ensembl.org/Mus_musculus/Variation/Explore?db=core;r=17:3448029-3449029;tl=OMfg43igjBvkvcNt-495810-71084463;v=rs33069377;vdb=variation;vf=5914270) |
| 21 | GTC - GTT | silent | *t*-specific |
