# Supplementary material for: Two isoforms of the RAC-specific guanine nucleotide exchange factor TIAM2 act oppositely on transmission ratio distortion by the mouse t-haplotype
Source: PLoS Genet. 2019 Feb 28;15(2):e1007964. doi: 10.1371/journal.pgen.1007964 (PMC6394906; doi:10.1371/journal.pgen.1007964)
Supplement: S2 Table — Structure of different complete and partial t-haplotypes and their transmission ratio distortion rates depending on the genetic background. (DOCX) [file pgen.1007964.s003.docx]

**Charron et al. Supplementary Table 2: *t*-haplotypes.** Structure of different complete and partial *t*-haplotypes and their transmission ratio distortion rates depending on the genetic background.

| **t-haplotype** | **structure** | **Reference, Transmission rate, (genetic background)** |
| --- | --- | --- |
| ***t^0^***  ***t^12^***  ***t^w5^***  ***t^w32^*** | 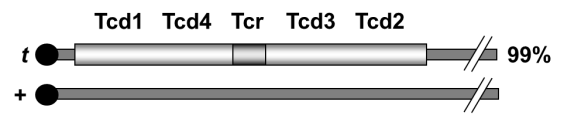 | [1] *t^0^*  34% (Ttf)  53% (BALB/c)  87% (BTBR/TF)  94% (CF1 )  [2] *t^12^*  52% BTBRTF/Nev.Ttf/t12  99% C3H/DiSn.Ttf/t12 |
| ***t^h49^*** | 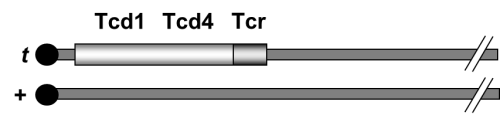 | [3] 39,8%  [4] 27%  [5] 47% |
| ***t^w18^*** | 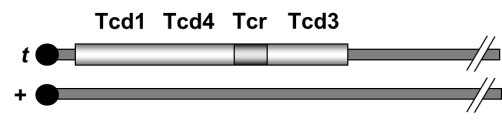 | [3] 55,8%  [4] 44,3% |

References

1. Bennett D, Alton AK, Artzt K. Genetic analysis of transmission ratio distortion by t-haplotypes in the mouse. Genetical research. 1983;41(1):29-45. PubMed PMID: 6840548.

2. Gummere GR, McCormick PJ, Bennett D. The influence of genetic background and the homologous chromosome 17 on t-haplotype transmission ratio distortion in mice. Genetics. 1986;114(1):235-45. PubMed PMID: 3770466.

3. Lyon MF. Transmission ratio distortion in mouse t-haplotypes is due to multiple distorter genes acting on a responder locus. Cell. 1984;37(2):621-8. PubMed PMID: 6722884.

4. Bauer H, Schindler S, Charron Y, Willert J, Kusecek B, Herrmann BG. The nucleoside diphosphate kinase gene Nme3 acts as quantitative trait locus promoting non-Mendelian inheritance. PLoS genetics. 2012;8(3):e1002567. doi: 10.1371/journal.pgen.1002567. PubMed PMID: 22438820; PubMed Central PMCID: PMC3305403.

5. Bauer H, Veron N, Willert J, Herrmann BG. The t-complex-encoded guanine nucleotide exchange factor Fgd2 reveals that two opposing signaling pathways promote transmission ratio distortion in the mouse. Genes & development. 2007;21(2):143-7. PubMed PMID: 17234881.
