# Supplementary material for: Two isoforms of the RAC-specific guanine nucleotide exchange factor TIAM2 act oppositely on transmission ratio distortion by the mouse t-haplotype
Source: PLoS Genet. 2019 Feb 28;15(2):e1007964. doi: 10.1371/journal.pgen.1007964 (PMC6394906; doi:10.1371/journal.pgen.1007964)
Supplement: S3 Table — (DOCX) [file pgen.1007964.s004.docx]

**Charron et al. Supplementary Table 3: Transgenic overexpression of *Tiam2l* decreases *t^h49^* transmission (Data from Fig 2D, E).**

(A)

|  |  | Offspring | | | | |  |
| --- | --- | --- | --- | --- | --- | --- | --- |
| Genotype of male | Number of males | *t* | + | total | % *t* | χ^2^ | P |
| *Tg1L/0; t^h49^/+* | 6 | 109 | 230 | 339 | 32 | 6.23 | 0.0125 |
| *+/+; t^h49^/+* | 6 | 181 | 259 | 440 | 41 |  |  |
| *Tg2L/0; t^h49^/+* | 6 | 92 | 312 | 404 | 22 | 4.35 | 0.0369 |
| *+/+; t^h49^/+* | 6 | 123 | 299 | 422 | 29 |  |  |

(B)

|  |  | Offspring | | | |  |  |
| --- | --- | --- | --- | --- | --- | --- | --- |
| Genotype of male | Number of males | *t* | + | total | % *t* | χ^2^ | P |
| *Tg1S/0; t^h49^/+* | 7 | 108 | 416 | 524 | 21 | 1.1 | 0.29 |
| *+/+; t^h49^/+* | 7 | 91 | 419 | 510 | 18 |  |  |
| *Tg2S/0; t^w18^/+* | 7 | 473 | 94 | 567 | 83 | 0.6 | 0.46 |
| *+/+; t^w18^/+* | 5 | 570 | 128 | 698 | 82 |  |  |

Abbr.: +, wild type; Tg1L, Tg(Tiam2)C1L1Bgh; Tg2L, Tg(Tiam2)C1L2Bgh; Tg1S, Tg(Tiam2)C11S1Bgh Tg2S, Tg(Tiam2)C11S2Bgh; 0 indicates hemizygosity.
