# Supplementary material for: Two isoforms of the RAC-specific guanine nucleotide exchange factor TIAM2 act oppositely on transmission ratio distortion by the mouse t-haplotype
Source: PLoS Genet. 2019 Feb 28;15(2):e1007964. doi: 10.1371/journal.pgen.1007964 (PMC6394906; doi:10.1371/journal.pgen.1007964)
Supplement: S4 Table — (DOCX) [file pgen.1007964.s005.docx]

**Charron et al. Supplementary Table 4: Heterozygous loss of *Tiam2* function strongly reduces the transmission rate of *t^w18^* (Data from Fig 3D).**

|  |  | Offspring | | |  |  |  |
| --- | --- | --- | --- | --- | --- | --- | --- |
| Genotype of male | Number of males | *t* | + | total | % *t* | χ^2^ | P |
| *Tiam2^LS/t^; t^w18^/+* | 7 | 241 | 183 | 424 | 57 | 43.984 | 0.0001 |
| *Tiam2^+/t^; t^w18^/+* | 7 | 378 | 109 | 487 | 78 |  |  |

Abbr.: +, wild type; *Tiam2^LS^*, *Tiam2^tm1Bgh^.*
