# Supplementary material for: Two isoforms of the RAC-specific guanine nucleotide exchange factor TIAM2 act oppositely on transmission ratio distortion by the mouse t-haplotype
Source: PLoS Genet. 2019 Feb 28;15(2):e1007964. doi: 10.1371/journal.pgen.1007964 (PMC6394906; doi:10.1371/journal.pgen.1007964)
Supplement: S5 Table — (DOCX) [file pgen.1007964.s006.docx]

**Charron et al. Supplementary Table 5: Transgenic *Tiam2s* overexpression strongly increases the transmission rate of *t^w18^* upon loss of the wild-type *Tiam2* allele (Data from Fig 3E).**

|  |  | Offspring | | |  |  |  |
| --- | --- | --- | --- | --- | --- | --- | --- |
| Genotype of male | Number of males | *t* | + | total | % *t* | χ^2^ | P |
| *Tg1S/0; Tiam2^LS/t^;t^w18^/+* | 7 | 244 | 237 | 481 | 51 | 13.4 | 0.0002 |
| *+/+; Tiam2^LS/t^;t^w18^/+* | 7 | 174 | 277 | 451 | 39 |  |  |

Abbr.: +, wild type; 0 indicates hemizygosity.
