# Supplementary material for: Two isoforms of the RAC-specific guanine nucleotide exchange factor TIAM2 act oppositely on transmission ratio distortion by the mouse t-haplotype
Source: PLoS Genet. 2019 Feb 28;15(2):e1007964. doi: 10.1371/journal.pgen.1007964 (PMC6394906; doi:10.1371/journal.pgen.1007964)
Supplement: S6 Table — Primer sequences, PCR conditions and results for transcript—and genomic analyses, generation of transgenics and gene targeting constructs. (DOCX) [file pgen.1007964.s007.docx]

**Charron et al. Supplementary Table 3: Oligonucleotides.** Primer sequences, PCR conditions and results for transcript - and genomic analyses, generation of transgenics and gene targeting constructs.

| **Experiment** | **Primer sequence (5’ - 3’)** | **Name** | **Number of cycles/ annealing temp.** | **Product size**  **base pairs** |
| --- | --- | --- | --- | --- |
| **Transcript analysis** |  |  |  |  |
| Amplification of *Tiam2l* cDNA | TACCGGTatgGGGAACTCCGAGAGTCAATATACC  GGATCGATTCATGATTTGCCATGGCTCTGTGTTTC | 5'-AgeI-Tiam2  3'-ClaI-Tiam2 | 10 cycles / 70 to 60°C  (-1°C/cycle)  30 cycles / 60°C | 5160 |
| Amplification of *Tiam2s* cDNA | TACCGGTAGTATGGAAGCACCAACAGAG  TAATCGATTCATGATTTGCCATGGCTCTG | 5'-AgeI-shortTm2  3'-ClaI-shortTm2 | 35 cycles / 60°C | 1860 |
| Generation of a *Tiam2ls* Northern blot probe | TACCGGTAGTATGGAAGCACCAACAGAG  GCGCCCTGCTCCAAAGGGATAGGTGCTAGTAC | 5'-AgeI-Tiam2short-2  TMFL ApaI as | 35 cycles / 55°C | 2100 |
| Generation of a *Gapdh* Northern blot probe | GGTGCTGAGTATGTCGTGGA  CACATTGGGGGTAGGAACAC | Gapdh-s  Gapdh-as | 25 cycles / 55°C | 220 |
| RT-PCR for *Tiam2l* and *Tiam2s* | TCCGGAAAGTCATCCAGGAGC  AGATGGTTTCTGGCCGTCCTTC | 5'-Tiam2-Ab2  3'-Tiam2-Ab2 | 35 cycles / 55°C | 999 |
| RT-PCR (*Gapdh* control) | ACCACAGTCCATGCCATCAC  TCCACCACCCTGTTGCTGTA | 5'-Gapdh  3'-Gapdh | 35 cycles / 60°C | 452 |
| *Tiam2s* transcript identification | TGAGCATGACCTTCCCTCTCTC  CAGTGACACTAGCTCCTTGAGAAGC | Hb853  Hb851 | 35 cycles / 55°C | 661 |
| *Tiam2s* amplification from first to last exon | TGAGCATGACCTTCCCTCTCTC  GATTTAGTCTTGTAATAAAATTGCATTTTAATT | Hb853  Tiam2_3’-utr-24 | 35 cycles / 55°C | 2202 |
| **RT q-PCR** |  |  |  |  |
| *Tiam2l* | ACCCTCAAAGCCAGAATGCG  TACTCACTGCCCTCCATGGA | Hb881  Hb882 | 40 cycles / 60°C | 110 |
| *Tiam2s* | TGAGCATGACCTTCCCTCTCTC  CATGGCTCTCTGTTGGTGCTTC | Hb853  Hb878 |  | 116 |
| *Gapdh*  (Ref 36) | TGTGTCCGTCGTGGATCTGA  TTGCTGTTGAAGTCGCAGGAG | Hb576  Hb577 |  | 150 |
| **Experiment** | **Primer sequence (5’ - 3’)** | **Name** | **Number of cycles/ annealing temp.** | **Product size**  **base pairs** |
| **Gene targeting** |  |  |  |  |
| Amplification of left arm of *Tiam2* targeting construct | TTATCGATTACCTGTATTCTCTGCAC  AAAAAGCTTTCTGGTCAGCGCTCTGAG | 5´-87  3´-88 | 35 cycles / 55°C | 2064 |
| Amplification of right arm of *Tiam2* targeting construct | AAGCTAGCACCCAAGATGAGGTAAAT  AACCGCGGACGCGTGCAGACCAGCCATTTAG | 5´-91  3´-92 | 35 cycles / 55°C | 3065 |
| Left (5’-) probe for detection of *Tiam2* homologous recombination | CTTGTGCTCAGGATTTTC  AAGGCCACAGTCTATGC | 5´-LP  3´-LP | 35 cycles / 55°C | 837 |
| Right (3’-) probe for detection of *Tiam2* homologous recombination | GGGAAACCTGGTGTGATGG  TCTGTGAGTTTCGGTCTAC | 5´-RP1  3´-RP1 | 35 cycles / 55°C | 919 |
| Genotyping for *Tiam2* wild-type (wt) and knock out (ko) allele | AACAAGTAGGCAGTCTCATTC  TAAAGCGCATGCTCCAGACTGCC | 5'-105  NeoAS | 35 cycles / 56°C | ko band: 501 |
|  | AACAAGTAGGCAGTCTCATTC  CCTCATAACCCAAAGTGATCT | 5'-105  3'-108 | 35 cycles / 50°C | wt band: 816 |
| **Transgenic construct** |  |  |  |  |
| Amplification of Ace promoter | AGGGCCCTTGGGGTCAGG  GGCCGCAGGAAAGCAGAG | Ace-s  Ace-as | 35 cycles / 50°C | 600 |
| Amplification of the ORF of *Tiam2* long | TACCGGTatgGGGAACTCCGAGAGTCAATATACC TAATCGATTCATGATTTGCCATGGCTCTGTGTTTC | 5'-AgeI-Tiam2  3'-ClaI-Tiam2 | 10 cycles / 70°C (-1°C/cycle)  30cycles / 60°C | 5160 |
| Genotyping for transgenic *Tiam2L* construct | AGGGCCCTTGGGGTCAGG  AGCTTTCCCAGGGTCTTGAT | Ace-S  3'-YC-Tg1-1 | 35 cycles / 50°C | 600 |
| ***t*-haplotype genotyping** |  |  |  |  |
| Genotyping for proximal *t*-haplotype region | TCATGGACCAACACAAGCTC  CACAAAACTGAAATCTCCCTCTC | vil2-L  vil2-R | 40 cycles / 57°C | wt band: 228  *t*-band: 195 |
